# Recollections on the Origins and Development of the Prosomeric Model

**DOI:** 10.3389/fnana.2021.787913

**Published:** 2021-12-24

**Authors:** Luis Puelles

**Affiliations:** Department of Human Anatomy, Biomedical Research Institute of Murcia (IMIB-Arrixaca), University of Murcia, Murcia, Spain

**Keywords:** Puelles biographic data, neuromeric models, columnar model, His, Herrick, Kuhlenbeck, Rendahl, Bergquist and Källén

## Abstract

The prosomeric model was postulated jointly by L. Puelles and J. L. R. Rubenstein in 1993 and has been developed since by means of minor changes and a major update in 2012. This article explains the progressive academic and scientific antecedents leading LP to this collaboration and its subsequent developments. Other antecedents due to earlier neuroembryologists that also proposed neuromeric brain models since the late 19th century, as well as those who defended the alternative columnar model, are presented and explained. The circumstances that apparently caused the differential success of the neuromeric models in the recent neurobiological field are also explored.

## Introduction

The present special number of BBEV titled ‘*Beyond the prosomeric brain model’* offers me the opportunity to present some notes explaining how I came to be involved in the birth of this model. I would not have space for reviewing relevant literature in too much detail, or even to mention all our publications using this brain model. I will rather highlight the major circumstances that, in retrospect, seem to have led me to this model. Although the account deals initially largely with my own experiences, the active participation of various colleagues and collaborators increases in importance afterward. The conception of the model was, in any case, strongly influenced by the published work of earlier scientists, or influential scientists I met, which also need to be mentioned and placed in context. I think that this story began when I first found neuromeric ideas at the school of medicine of Granada (Spain), in 1971, as a recent postgraduate. I was photocopying selections from the Journal of Comparative Neurology.

## First Impression of a Neuromeric Model

During the last year of my medical studies at the University of Granada (term 1970–1971), I started to attend the embryological laboratory of a newly incorporated professor of anatomy, Génis-Gálvez (1924-2003; see biographical notes on Génis-Gálvez in Velasco-Morgado, 2014). He had transferred from Salamanca to Granada in 1968. A few years before, I had passed my anatomy and embryology subjects with a different professor. He, therefore, did not teach me at all. After 3 years of preclinical subjects and two 2 of clinical studies, I had decided to finish my sixth and final year of medicine studies, but *not* to become a practicing clinician, for which I had found I had little vocation. I had chosen instead a research career in neurobiology, preferably basic. Understanding the mind had been my major interest since adolescence, when I first tried to explain to myself volatile adolescent amorous reactions observed within my school class (boys and girls half and half). However, I was detoured from my consequent strong interest in psychology (then only approached in Spain as a third year subject in medical studies) by the dogmatic verbiage, devoid of any connection with the brain, offered by my psychology and psychiatry professors in Granada (third and fourth terms). At the start of my last term, I had already decided to attempt a formation in neurobiological research. After short visits of the physiology and histology/pathology departments (where I found no neurobiological research was done), a professor suggested to try the ‘new’ anatomy professor J. M. Génis-Gálvez, who did embryological research on the eye. The idea was to occupy myself with brain development during the last year of medicine and then apply for entrance at a leading neurophysiology laboratory in Madrid.

Professor Génis-Gálvez was very receptive. He explained to me mainly the possibilities to do experimental embryology in his lab. To this end, he showed me in the lab how one opens an incubated egg and finds inside the chick embryo, with its big eyes, beating heart, and transparent brain vesicles. It was love at first sight. Obviously, this morphological approach with experimental possibilities relegated to the far background my psychological and physiological interests on the mind, although they persisted up to the present as a reading hobby. I thought, though, that psychology apparently had nothing to offer me at that time point. It seemed that there was a lot of preliminary work to be done yet on basic brain structure before higher mental properties could be properly addressed. Moreover, I estimated that the status of neurophysiology probably was not much more advanced in this sense than that of psychology. I, thus, came out of the interview with a handful of reprints on brain development to read and a very vague idea that I wanted to study brain structure, hoping to develop more specific plans as I advanced. I read immediately the articles Génis-Galvez had given me, sitting in a chair just outside his door, and entered his office again 20 min later to ask him for more. This must have been in October 1970. I was then 21 years old.

My tutor worked specifically on the developing eye (he was a non-practicing ophthalmologist originally), and thus could not teach me much about the brain proper beyond what one learned then in medical neuroanatomy. However, he was happy to have in the lab somebody who would try to develop a line of work in central aspects of the visual system. He helped me by providing guiding literature (he had a good collection of basic books, some journals, and reprints obtained during his stays in the States), and placed at my disposal a small laboratory, a technician, chick eggs, and any chemicals I needed. I started to work away with maximal dedication and dropped altogether attending my last term classes (I just crammed the subjects 3–4 days before the exams).

Normally I read in my free time a book from Génis-Gálvez’s library every week, until I read most of them. These readings introduced me particularly to modern *cell and developmental biology* including histology, electron microscopy of cellular fine structure, genetics, biochemistry, molecular biology (I remember particularly the book by Watson on ‘*The Gene*’), and descriptive and experimental embryology. Some of the books touched specific aspects of nervous system development (e.g., neurons in culture, axonal growth cones). I hardly read anything about adult neuroanatomy proper until I started to teach the subject some years later, although I worked occasionally on my copy of the two-volume treatise of Ramón y Cajal, his ‘Histologie du Système Nerveux de l’Homme et des Vertébrés’ 2nd edit (Ramon y Cajal, 1909, 1911). My intense readings during that first year represented an introduction to modern biology, although without systematic botany or zoology, thus, complementing partially my standard medical formation in a direction helpful for research. I cannot say that I learned much about the brain, except what I saw personally at the microscope. The theory of evolution apparently was not studied at all in Granada at the time. I learned of its major conclusions only years later in books.

At the beginning, I thus had no idea of *comparative* neuroanatomy or neuroembryology. I simply extrapolated what little I knew from the human brain to the chicken brain. I assumed that birds moved their eyes with comparable muscles and nerves, as Ramón y Cajal seemed to imply in his treatise. He was a pragmatic evolutionist and comparative morphologist, accepting *a priori* that vertebrate brains were comparable, in general, but he never discussed theoretical issues such as the concept of homology, brain organization models, or Haeckel’s and Baer’s contrasting ideas on developmental recapitulation of evolutive change. In the lab, there was no textbook on comparative neuroanatomy available nor an avian brain atlas. We accordingly just supposed that, at least as regards the visual system and oculomotor mechanisms, *equivalent structures* could be expected in humans (mammals) and birds. Fortunately, this assumption was right, as I learned afterward, but my initial rate of advance in learning to recognize chicken brain structures was extremely slow. The brain I had in front of me at the microscope was a large complex of structures I could not identify, with only a small area illuminated by understanding at the oculomotor nuclei. I needed many years of solitary toiling with reprints or photocopies and the microscope before I slowly expanded my neuroanatomic knowledge to neighboring areas, helped by avid exploration of literature on the brain of birds compared to those of other vertebrates. Much later, I expanded to the whole brain thanks to the prosomeric model, which allows prediction of structures you are going to see.

The end of such initial lack of information was reached some years later, when I found the physiologically oriented volumes of Laget’s ‘Éléments de Neuroanatomie Fonctionelle’ (Laget, 1972, 1973, 1976), which I bought in a visit to Paris while I was working in Sevilla (next section). This not particularly famous work introduces every brain portion with comparative anatomic summaries and schemata through all vertebrates, plus crucial citations from the comparative literature. I learned there about the existence of the classic three-volume treatise of Kappers et al. (1936) on ‘The Comparative Anatomy of the Nervous System of Vertebrates,’ which I immediately obtained. I finally found in these volumes a systematic source of comparative data on the avian (and other) brains and accordingly explored the classic comparative theory of the brain, led unknowingly by the columnar model of the brain based on the work of Herrick (1910) used in that treatise.

I, therefore, was not initially aware of the existence of alternative *models* of brain morphologic structure, based on either adult or embryonic data. Neuroanatomy textbooks, as a rule, do not mention the brain model they follow, possibly because brain dissection underlying neuroanatomy leads you to think in terms of apparent *facts*, rather than to conceive hypotheses, conjectures, or tentative assumptions on the data, and, accordingly, to organize knowledge around conceptual models (nevertheless, every description necessarily entails a supporting model, held consciously or not). It is only in recent years that neuroanatomy textbooks are starting to appear that emphasize their implicit theoretical model. These are only those based precisely on the *prosomeric model*, probably due to my emphasis on this important aspect (e.g., Watson et al., 2010; ten Donkelaar et al., 2018; Schröder et al., 2020; ten Donkelaar, 2020; new edition of the Benninghof treatise, in preparation).

The *columnar model* defended by Herrick (1910; 1933; 1948) and Kuhlenbeck (1927, 1973) was then absolutely prevalent and was implicitly assumed by all neuroanatomy book authors. All of us (experts and beginners alike) used that model, often without being aware of its existence or its fundaments (more on this model below). It is like breathing without knowing that air exists.

Over the summer of 1971, after finishing my medical studies, I returned to my parental home in Tenerife (Canary Islands) and married my girlfriend from preuniversitary studies and subsequent lab collaborator in Granada Margaret Martínez-de-la-Torre. After our honeymoon, I read the well-illustrated textbook of Embryology which had been published by Génis-Gálvez (1970). This stimulating book confirmed my interest in embryology, as well as in its experimental possibilities. In September, we moved back to Granada, rented an apartment, and I started to work on my doctoral thesis project (officially tutored by Génis-Gálvez, although he just let me do what I wanted). I also began to teach some anatomy and dissection for medical students as a part-time teaching auxiliary, earning very little. Fortunately, we had economic support from my parents, who always confided in me, although it was clear to all of us that a research career in the Spain of Franco was highly risky.

In this period, my tutor applied for a transfer closer to his birthplace in Cadix, namely, to a position at the anatomy department in the University of Sevilla (Granada, Cadix, and Sevilla, where I worked in my formative years, are all Andalusian cities; Sevilla, the Andalusian capital, is the largest of them; its university also encompassed then the isolated medical school at Cadix). Génis-Gálvez obtained the Sevilla position, and in his plans for transferring in 1972 the whole lab (including Margaret and me), he got funding from the Granada faculty dean to photocopy any departmental journal article that was of interest to us, since the journals had to be left behind. The job to decide which articles were to be photocopied in the Journal of Comparative Neurology fell to me (we had an ample collection). For weeks, I thus spent hours perusing systematically through hundreds of JCN articles, just examining them superficially, since I really knew little of such contents and nothing at all about the possible fame of the authors. If I found minimally interesting the title, abstract, figures, and/or schemata, I jotted them down for copying.

**FIGURE 1:**
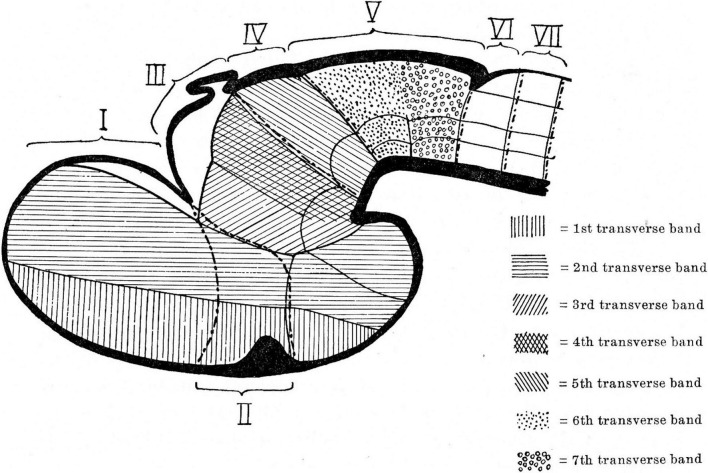
Neuromeric model of Bergquist and Källén (1954). Note that neuromeres I–VII are superposed by transversal bands 1–7. Also, neuromeres I–II imply an ending of the forebrain axis in the telencephalon (a columnar notion departing from the axial concept of His). In subsequent publications, Bergquist and Källèn corrected this error (see Figure 6), noting that neither the telencephalon nor the eye vesicles represent true neuromeres.

Curiously enough, among hundreds of JCN papers inspected in Granada, the only article whose images stayed in my memory was that of Bergquist and Källén (1954), a work in which these Swedish authors, then fully unknown to me, presented internationally their *neuromeric model of the brain of vertebrates*. This was actually my first exposure to a topologic brain map and its deep theoretical fundaments discussed in the corresponding text. Without really understanding fully most of what the authors said, or what it implied, I felt a strong emotional response of interest to the schemata. For some unconscious reason, this apparently topologic approach representing the brain and its subdivisions in a flattened schematic form was *highly interesting* to me. I still remember *exactly* where I was and how I stood there with the 1954 number of JCN in my hands, looking at those schemata, and even can visualize the main schema itself, exactly as it appeared in the page (Figure 1). It was a totally unexpected impact and, moreover, not a result of understanding the need for such a schema, but of just noting *a new possibility to visualize synthetically brain structure!* It was the sort of spine-tingling emotion produced otherwise by sublime musical passages which one does not understand technically. The neuromeric article thus passed to the collection we brought to Sevilla. However, subsequent calm reading did not show how to relate those interesting theoretical ideas to my daily work in the lab on the oculomotor nuclei and the interstitial nucleus of Cajal (the subject of my thesis). The needed context of being aware of alternative brain models and differential explanatory capabilities was wholly absent. I thus, left the neuromeric model aside, without forgetting the impression it had made on me, and only returned to it several years later when its implicit content started to become meaningful.

## Period in Sevilla (1972–1976)

In Sevilla, I obtained a 3-year predoctoral fellowship that resolved our economic situation. I finished in 2 years my autodidactic doctoral thesis on the quantitative development of the population of the interstitial nucleus of Cajal in 1973 (Univ. Sevilla), although I never showed it to anybody due to the methodologic shortcomings I saw in it. Later, I published a paper on the migration across the midline of part of the oculomotor nucleus population (Puelles-López et al., 1975). This was my first publication, the only one in which I joined my paternal and maternal family names. In the summers, I did several small stays in Paris (in 1974 and 1975) helped by a fellowship from the European Training Programme in Brain and Behaviour Research. I visited the lab section of Alain Privat on organotypic brain culture at the INSERM U106. My project there was to learn organotypic culture methods, aiming to reproduce *in vitro* the oculomotor migration I had identified in the chick in order to make it accessible to experiments. However, the approach did not produce significant results. Since Privat normally worked with rat embryos, we resolved to check whether the oculomotor migration also occurred in that species analogously as in my published report on the chick. We used neurofibrillary and electron-microscopic methods (for a closer look at possible cell contacts and cytoplasmic cytoskeletal details). Our resulting publication (Puelles and Privat, 1977) was the first demonstration of the crossed oculomotor migration phenomenon in mammals and represented my second publication, as well as my first experience with mammalian embryos.

A major neurohistological figure I met at the INSERM U106 was Constantino Sotelo, an expatriated terminal member of Cajal’s school (direct student of Fernando de Castro, one of Cajal’s younger collaborators). He was then famed as one of the major experts in brain electron microscopy and also was interested at the time in neuroembryological questions. He recommended trying Golgi impregnation methods on the oculomotor migration and particularly the variant of Stensaas (with glutaraldehyde substituting osmium tetroxide in the fixative solution), which he thought worked better in embryos than the standard rapid Golgi procedure (osmium fix). I, thus, started a collection of Golgi-Stensaas impregnated rat embryos, which I brought with me to Sevilla at the end of the stay. Back home, I also tried out the Stensaas procedure on chick embryos and found it worked much better than in rats (unless the exceptional success was due to the water of Sevilla).

## Histogenetic Studies: Golgi Studies on Early Neuronal Sequences of Differentiation in the Midbrain Tectum (1974–1977)

During the 1974/1975 term in Sevilla, I concentrated on the increasing collection of chicken Golgi preparations. Apart from my teaching duties (several hours daily), I generally processed one embryo per week (fixation, reaction, embedding, cutting, and mounting the thick celloidin sections strictly ordered on slides, and finally looking at them at the microscope). Beautifully impregnated developing neurons and many other sorts of cells appeared filled up by the reddish-brown silver chromate precipitate. I remember spectacular renal podocytes, as well as osteoblasts, vascular endothelial cells, fibroblasts, mastocytes, etc., apart from the desired neuronal and glial cells.

**FIGURE 2:**
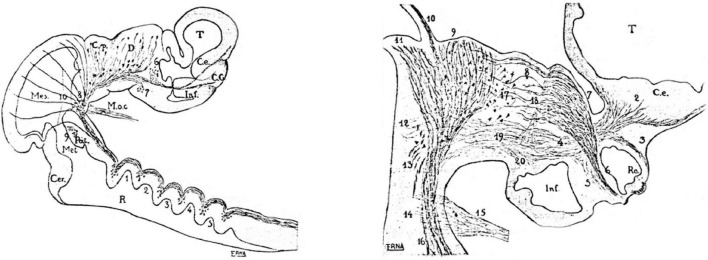
Selected neuromeric schemata from the work of Tello (1923), showing sagittal sections through chick embryo brains at two different stages, wherein diverse reduced-silver-stained tracts clearly relate to neuromeric bulges. On the right, the growing optic tract is represented as a longitudinal tract relative to the diencephalic neuromeres.

I first spent many hours just gazing at this histological spectacle, since I had no clear objective in mind. Eventually, due to our general interest in the visual system and the published Golgi work of the Cajal brothers (Ramon y Cajal, 1891, 1911; Ramón, 1898, 1899, 1943) on the adult neuronal types and cell layers found in the avian optic tectum, I concentrated on a Golgi study of the differentiation and histogenesis (migration and stratification) of the reported tectal cell populations. For guidance, I had some Golgi results of Leghissa (1957, 1958) on the tectum of chick embryos older than 9 days *in ovo* and an extensive description of early tectal neurofibrillar development by Cajal’s collaborator J. F. Tello (Tello, 1922, 1923). This material indicated when the earliest neurons appeared (Figure 2). The aim was to follow the example of Cajal on the developing spinal cord, retina, and cerebellum (see my later review on embryological work of Cajal and Tello in Puelles, 2009). I, thus, became an autodidact distant pupil of the school of Cajal.

I also collaborated with Génis-Gálvez in a study of the development of inverted retinal amacrine cells, contributing some of my Golgi-impregnated images (Génis-Gálvez et al., 1977). This was the first time we used tritiated thymidine autoradiography, a technique brought to us by a younger member of the Sevilla lab, my good friend Carmen Prada, who did a summer stay in the lab of I. Smart in Dundee (Scotland). We later did two additional Golgi papers on the developing chick retina with C. Prada as first author. I also inserted some of my own retinal Golgi data in Puelles (2009).

The school of Cajal had practically disappeared in the post-civil-war period and only the publications remained. Cajal died in 1934, Cajal’s brother Pedro (P. Ramón), who was active in his youth in comparative brain studies, moved to clinical work in Zaragoza, and Tello (the only pupil of Cajal doing neuroembryology) died in 1958, having been expelled from the direction of the Cajal Institute in 1936, as a collateral result of the Spanish civil war. I received some technical advice and psychological support from Constantino Sotelo, as well as his personal example, since he was the first scientist of excellence I met. Sotelo also supported my ulterior first publication on the chick neuromeric model (see below).

Interestingly, in contrast to Cajal, who never mentioned neuromeres, Tello used *neuromeric concepts and schemata* in his descriptive neurofibrillary studies of the chicken and mouse embryonic brains (Tello, 1922, 1923, 1934; see biography of Tello in Collazo Rodríguez, 1981). However, he did not explain why he chose this model (or which sources he used); he just correctly delimited diencephalic and rhombencephalic neuromeres as visible developmental landmarks for given developing tracts and neuronal groups (Figure 2). Cajal, who most probably read and approved these papers of Tello, apparently did not object to the use of the neuromeric concepts. I duly noticed this neuromeric aspect in the developmental work of Tello (1922, 1923, 1934), which preceded in time the work of Bergquist and Källén (1954, cited above, Figure 1) and also slightly preceded the neuromeric model of Rendahl (1924), another important antecedent, commented below. The neuromeric papers of Tello were the second place where I encountered neuromeric concepts, although at this point I was concentrated on cellular differentiation details of the tectal neurons and was not attending at all to the issue of neuromeres.

The problem posed by the tectum (a large rostral alar midbrain domain) was difficult because there are some 14 distinct tectal neuronal cell types whose differentiation sequences and stereotyped layering patterns had to be explained (tectal layering was known to change substantially in evolution; Kappers et al., 1936). However, a positive aspect was that the large avian optic lobe has a strong anteroposterior developmental gradient (as had been just published in the very useful autoradiographic neurogenetic studies by LaVail and Cowan, 1971a,b). This gradient allows tentative differentiation stages to be checked rostralwards (where more advanced cells can be seen) and caudalwards (where less advanced cells are found), as long as the preparations are as homogeneously Golgi-impregnated as mine were. This project took most of my research time during my last years in Sevilla (up to the end of the 1975/1976 term, in September), and I finished the paper in Badajoz, where we moved for the next term. I left aside momentarily the oculomotor migration problem.

**FIGURE 3:**
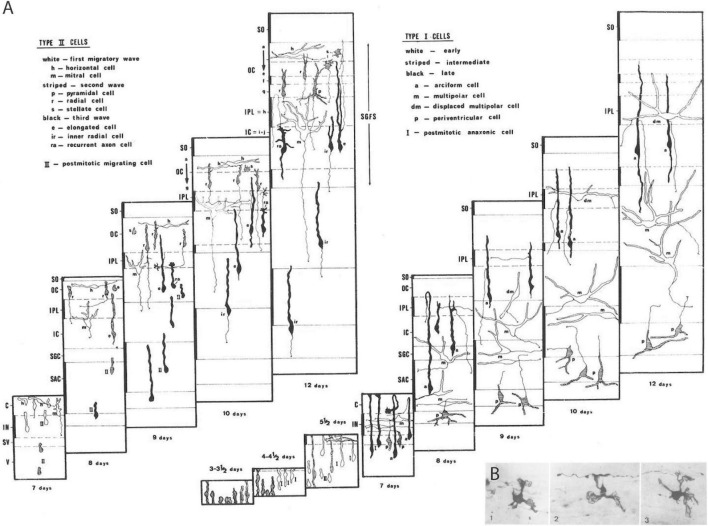
Examples of Golgi results on neuronal and glial differentiation patterns. **(A)** Unpublished schema from the thesis of Bendala (1978), collecting all results on tectal cell types I and II in the context of developing tectal layer formation from Puelles and Bendala (1978). The time range covers 3–12 days of incubation. **(B)** Example of immature oligodendrocytes (from the chick optic tectum) displaying a small number of velamentous immature processes that are starting to envelop passing axons, revealing presumably the first step in the formation of myelin sheats (from Puelles, 1978a).

Having used the 1974/1975 academic term preparing the chick Golgi collection, I spent the whole summer of 1975 alone in the lab, toiling away for 12–14 h a day in the extreme Sevillian heat (no air-conditioning), studying the data. I drew tectal cells one after another with a *camara clara* device, noting the layers they appeared in, and photographed the best of them (I took a good number of photographic rolls every day, which I processed to positives in the afternoons). There was a stimulating ‘explorer’ feeling: I saw myself as the first person ever *to look* at these incredible tectal histogenetic phenomena (how young tectal neurons migrated, how they produced their dendrites and their axonal outgrowths, and how they stratified differentially according to the two different neuroblast prototypes I soon discovered, which I called Type I and Type II cells; Figure 3).

There were glorious moments at the microscope, when sudden flashes of insight occurred, with tingling running along my spine (e.g., when I distinguished free neuroblast migrations guided by radial glia from cases of somatic translocation, both occurring side by side in my material; free neuroblast migration was not accepted as possible at the time, for example, in the work of Morest, the major expert). Whole sets of observations were sometimes explained in a flash in terms of a particular novel cellular behavior or differentiation sequence. I used these insights to generate predictions that often were triumphantly verified in the tectal gradient. There was a large blackboard in the departmental corridor, where I gradually drew my conclusions on a stage-by-stage map of tectal layers, separating Type I from Type II cells. When Génis-Gálvez returned from his vacation in September, he was amazed to see the whole blackboard squirming with subtly changing cell shapes (Figure 3A).

During the 1975–1976 term, the tectal work advanced at a slower pace into later stages of development (9–12 days of incubation) with the help of a doctoral student and thereafter also dear friend, biologist Carmen Bendala. We jointly discovered an additional more retarded Type III sort of tectal cell prototype, which typically displayed *ascending* axons. She read her thesis on the chick tectum in 1978, the year our publication came out, when I was already in Cadix after spending a year in Badajoz. However, most of the late tectal data remained unpublished.

For the 1976–1977 term, I unexpectedly was offered a contract as full professor and director of the anatomy department at the University of Extremadura (at Badajoz, next to Portugal). I decided to accept it, although this implied a transient rupture with Génis-Gálvez, who wanted me to stay in Sevilla. As a result, my Golgi collection had to remain in Sevilla. I, thus, started a new laboratory and a new Golgi collection in Badajoz, where I was wholly on my own as regards research and the sort of neuroanatomy I taught. I prepared there under singular conditions (no funding) the manuscript on early differentiation of tectal cell types.

The paper was published in 1978 in Neuroscience, after small adjustments (Puelles and Bendala, 1978). The tectal neuronal migration and differentiation data we described were later largely corroborated in subsequent studies using more modern techniques, notably by the American Sanes and his colleagues (Gray et al., 1988, 1990; Galileo et al., 1990; Gray and Sanes, 1991, 1992; Leber and Sanes, 1995). We also were able to check subsequently that exactly the same tectal histogenetic pattern and cell types occur in the case of the developing lizard optic tectum (Baez et al., 2003; I co-supervised this doctoral thesis). A comparable result has not been produced yet in anamniotes or mammals, although some of the published Golgi data in fish, amphibians, and mammals show neuronal examples comparable to our cell types, and the optic tectum is known to be highly conservative in its connections. In mammals, some added elements (perhaps novel cell types) might occur, given what we know of divergent superficial tectal stratification and the massive cellular development of the periaqueductal gray compared to that of sauropsids and anamniotes. The periaqueductal gray is often figured as if it was independent from the superior and inferior colliculi, but its cells underlying the colliculi arise late from the same progenitor domains.

I think the Puelles and Bendala (1978) tectal report was my first important scientific publication (7 years after I started). In its treatment of all tectal cell types, it is the most complex histogenetic analysis ever done with the Golgi method. I am proud that it was performed without other guidance than the rationale exemplified previously by Ramón y Cajal, Tello, and Leghissa. This study taught me what sorts of differentiative and migratory complexities may be found in the histogenesis of a distinctly delimited progenitor area over time. The three distinct postmitotic neuron prototypes diversifying gradually into the various final neuronal forms strongly suggested that the genetic profile present at cell birth was highly important in determining the fundamental behavior of the cell, whereas the changing surroundings over time probably exert an epigenetic mechanistic modulatory role on the emerging phenotypes.

Another paper I submitted jointly to Neuroscience while in Badajoz dealt on the earliest Golgi-impregnated shapes of developing tectal oligodendrocytes (Puelles, 1978a; Figure 3B). It appeared slightly before the tectal one because it was accepted *without any changes*; this is the only time this happened to me. I think that these results still represent the only published visualization so far of young postmitotic oligodendrocytes in the process of enveloping axons with their lamellipodial processes, the future myelin sheets.

It was during this term in Badajoz, working on the tectum, that I obtained part of the multi-volume comparative treatise of Kuhlenbeck (published by Karger between 1967 and 1978; ‘The Central Nervous System of Vertebrates’), which I also studied with enormous interest. I found in this erudite German author, who died recently, a remarkable new virtual teacher, with whom I have had many mental discussions over the years. Unfortunately, I never met him.

The third volume, part II of the treatise of Kuhlenbeck, on ‘Overall morphologic pattern’ (Kuhlenbeck, 1973), elaborates a crucial *developmental* basis for the comparative analysis of brains which I found highly significant as a general procedure. Irrespective of my admiration, I came to disagree with Kuhlenbeck on some important details, particularly his notion that neuromeres are merely transient structures, and his support of a straight forebrain length axis ending in the telencephalon (in this he disregarded the cephalic flexure). I have since then kept this volume 3, part II, at my side for frequent consultation, and I recommend reading it to everybody in the field. It is very well written, as are all works of Kuhlenbeck, including his philosophical ones (he was also doctorated in philosophy). Importantly, his ‘Overall morphologic pattern’ volume also contains a detailed discussion of various developmental and non-developmental *brain models*, including Herrick’s (1910) *columnar model*, which was always supported by Kuhlenbeck, and the alternative *neuromeric models* of some German authors such as Von Kupffer (1906), Ziehen (1906), and Haller von Hallerstein (1929, 1934), and the Nordic school (see below).

**FIGURE 4:**
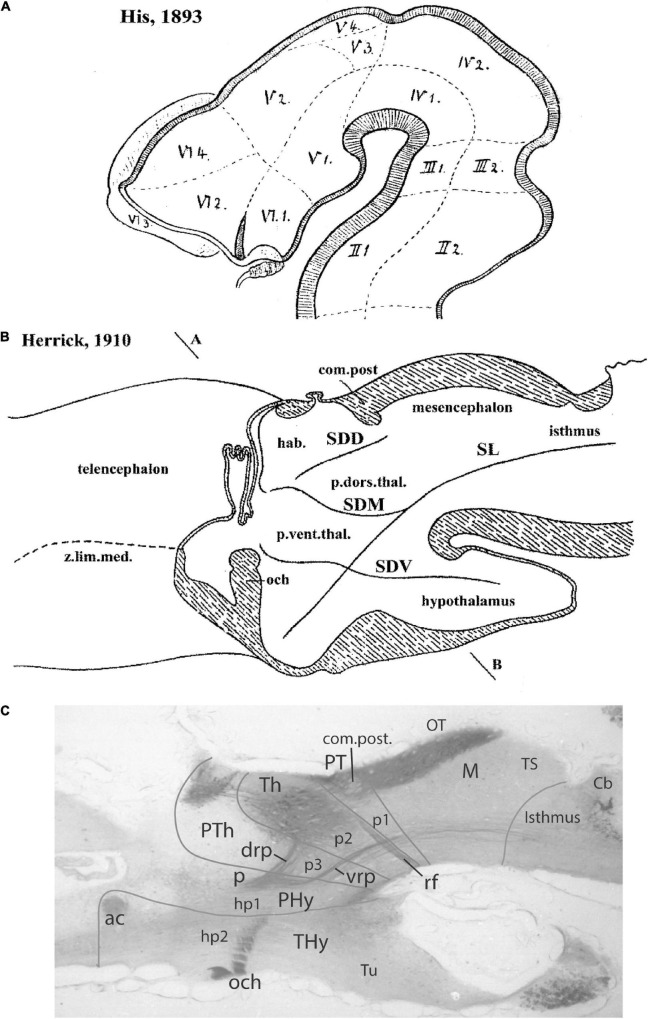
Comparison of the forebrain models of His (1893) **(A)** and Herrick (1910) **(B)** with a calretinin-immunoreacted sagittal section of the axolotl *Ambystoma tigrinum* (**C**). **(A)** The schema of His emphasizes his longitudinal alar-basal boundary, which correlates with the sulcus limitans that represents the length axis, ending in the hypothalamus. Note its bending around the cephalic flexure. Other boundaries depicted are more or less orthogonal to the axis, separating what he considered to be transverse elements of the brain. The telencephalon was interpreted as placed dorsal to the hypothalamus. **(B)** The columnar model of Herrick based on the axolotl salamander *Ambystoma tigrinum* shows that the three diencephalic sulci—SDD, SDM, and SDV—which he arbitrarily defined as landmarks that delimit ‘longitudinal’ columns in the diencephalon, actually cross obliquely the sulcus limitans of His (SL). By entering the SL into his schema, Herrick revealed the conceptual discrepancy about what should be assumed to be ‘longitudinal,’ which he left undiscussed. **(C)** Calretinin neuronal immunoreaction in the thalamus of *Ambystoma* (Th in p2), leaving unlabeled both the prethalamus (PTh; p3) and the pretectum (PT, p1); gray lines mark the transverse interneuromeric boundaries, according to our updated prosomeric model. This image belongs to the material prepared during the visit to the laboratory of Northcutt in 1992, which served to identify diencephalic prosomeres in this species. Note the caudal boundary of the thalamus is parallel to the *transverse* retroflex tract (rf) and all interneuromeric borders are orthogonal to the *longitudinal* dorsal and ventral roots (drp, vrp) of the cerebral peduncle (p), which collects into a single *transverse* bundle in the peduncular hypothalamus (PHy). The optic tract is seen ending in the midbrain optic tectum (OT). All these tracts were of course observed by Herrick (1910), but he interpreted them as coursing oblique to his axis.

**FIGURE 5:**
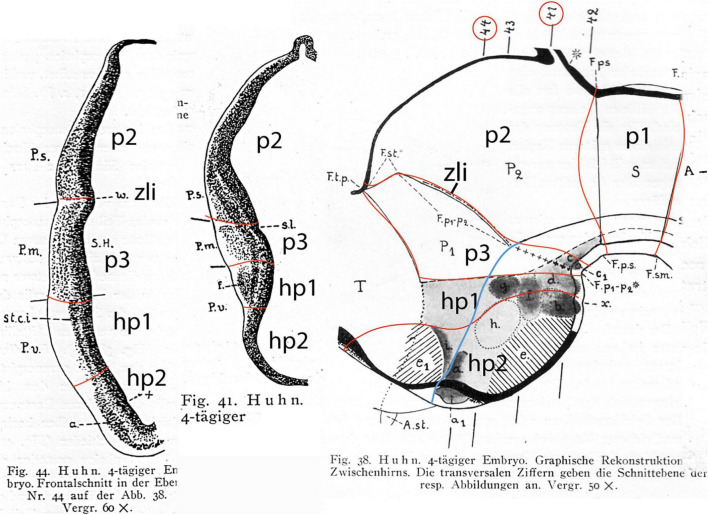
Drawings extracted from Rendahl (1924), showing his reconstructed neuromeric model of the chick diencephalon at 4 days of incubation and histological details in two cross-sections. I added the red lines and the large labels **p2**, **p3**, **hp1**, **hp2** to show comparatively where we place in our present updated model the neuromeric boundaries, largely coinciding with Rendahl. The p1 alar domain corresponds to the pretectum, whereas p2 and p3 mark the thalamus and prethalamus alar domains, respectively (note the neuromeres are wedge-shaped because they are deformed by the axial cephalic flexure). The p2/p3 boundary is the zona limitans intrathalamica, first defined by Rendahl, although it had been depicted by Tello (Tello, 1923; number 8 in the right part of Figure 2). The hp1 and hp2 domains are the two hypothalamo-telencephalic prosomeres postulated by us in the updated prosomeric model. The two cross-sections at left correspond to the section levels marked in the map as 44 and 41. They illustrate the differential histogenetic patterns and the abrupt boundaries corresponding to the interneuromeric borders, even within the hypothalamus, where Rendahl did not postulate neuromeres. He only represented the longitudinal sulcus limitans at p1 and p2, separating their alar and basal portions. We extend this landmark through p3 and the hypothalamus as well, ending under the optic chiasma (blue line).

**FIGURE 6:**
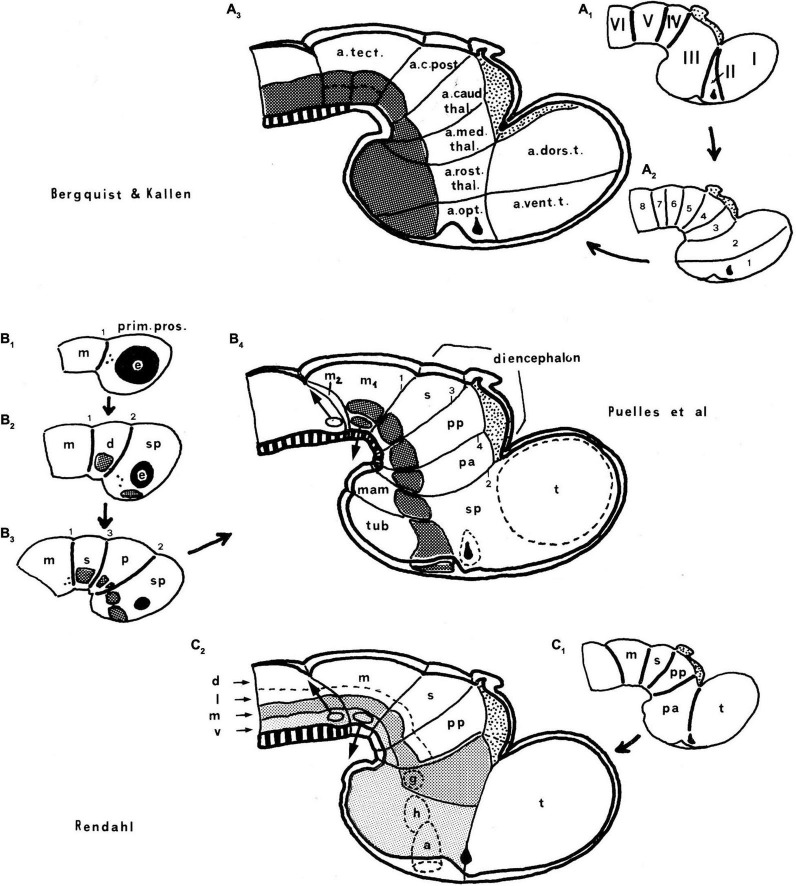
Comparison of the neuromeric models of Bergquist and Källén (1954) **(A3)** and Rendahl (1924) **(C2)** with our own chick model, extracted from Puelles et al. (1987a; **B4**). The precociously populated basal plate neuromeric modules are emphasized. The B&K schemata show in **(A1)** their neuromeres (later corrected) and in **(A2)** their transversal bands, which we take as corresponding most realistically with our neuromeres. Our schemata include early stages in the emergence of the neuromeric pattern in the schemata **(B1–B3)**. The Rendahl schemata **(C1,C2)** reveal that this author postulated the telencephalon as a prosomere I lying rostral to the diencephalon, thus showing the influence of the columnar axis. On the whole, it is clearly observed that the three sets of schemata are basically comparable in many aspects, irrespective of minor differences.

Kuhlenbeck strived since the early 20s to provide a consistent comparative embryologic basis for the initially adult version of the columnar model presented by Herrick (1910; Figure 4A). However, in my opinion he failed in this aim, as did Herrick on the whole, largely because *they both disregarded analyzing objectively and causally the brain axis*. The brain axis of the columnar model is an *imaginary arbitrary construct* produced by someone that is not interested in embryonic processes. I believe that no strong model can be constructed on this basis, due to unrecognized false assumptions. This contrasts with the essentially correct brain axis defined *beforehand* by His (1893) on the basis of neurogenetic heterochrony between the basal and alar plates, which was first recognized by him (Figure 4B). These concepts of His were later strongly corroborated by gene expression patterns and experimental embryology, substantially supporting also the prosomeric model. Neuromeric models usually refer to the length axis concept of His (1893, 1904), although sometimes the axis wrongly slides over from the hypothalamus into the telencephalon (e.g., Rendahl, 1924; Bergquist and Källén, 1954; Figures 1, 5, 6). This was caused by confusing the telencephalic and eye exclusively *alar evaginations* with complete neuromeres, which are characterized instead by the presence of floor, basal, alar, and roof longitudinal domains (Puelles et al., 1987a).

In any case, Kuhlenbeck (1973) presented and criticized the *neuromeric models* of Rendahl (1924), Haller von Hallerstein (1929, 1934), Bergquist and Källén (1954), and Vaage (1969). He generally reached the conclusion that initially existing neuromeric structure was later substituted by the development of Herrick’s columns in the mantle zone *across the interneuromeric boundaries*. This conclusion has been refuted by more modern descriptive and fate-mapping evidence (indeed, the original neuromeric limits can be visualized in the adult brain in transgenic mice). The Bergquist and Källén (1954) neuromeric notion which impressed me so strongly in 1971, thus reappeared again in my line of sight in 1976/1977, now in a more complete theoretical context, thanks to Kuhlenbeck. These ideas soon acquired added meaning, particularly as reflected in the monograph by Rendahl (1924; Figure 5).

## Golgi Study of the Diencephalon in Cadix, Initial Neuromeric Work With Ache (1977–1979), and the Neuromeric/Columnar Controversy

At the next term (1977/1978) we moved again, this time to the school of medicine at Cadix, where I was offered an adjoint full professor contract by José-Maria de Castro, a former lab companion and friend in Granada (also a pupil of Génis-Gálvez, several years older than me; he probably caused directly or indirectly the earlier offer of the professor position in Badajoz). José-María guided my efforts to obtain a tenured academic position (first as associated professor in Cadix [1978], and later as adjoint full professor in Murcia [1979]). In the meantime, he offered me a better laboratory infrastructure and funding than I had in Badajoz and full research liberty. I also got my own group of medical students to teach neuroanatomy and neuroembryology. Having to teach neuroanatomy daily is the best way to learn it yourself. I had started doing so already in Sevilla in 1973. The notes of the lessons I gave in Cadix were the skeleton for our subsequent neuroanatomy textbook, which was based of course on the prosomeric model (Puelles et al., 2008).

I had checked in Badajoz whether the three tectal neuron prototypes were generally present in other parts of the brain. They were not, as was already suggested by existing Cajal data on the spinal cord, retina, and cerebellum, substantiating the conclusion that the brain wall was apparently divided into multiple areal progenitor units with differential histogenetic patterns and characteristic cell types (a concept we later used in the prosomeric model, adding a differential molecular profile). Given the success of the Golgi study of the optic tectum, I chose to begin in Cadix a Golgi study of neuronal differentiation sequences in the developing chicken diencephalic visual centers. The latter were scattered over the hypothalamus, ventral thalamus, dorsal thalamus, and pretectum according to the columnar model. I was helped in this project by a new thesis pupil, biologist Cristina Zavala.

For the diencephalon, we initially applied the widely prevalent *columnar model* to the analysis of our Golgi data. We followed the columnar embryologic Nissl studies on chick diencephalic development done by Kuhlenbeck (reviewed in Kuhlenbeck, 1973). According to the interpretation of this author, the diencephalic columns, like their hindbrain counterparts, were cellularly *homogeneous* longitudinal masses of neurons displaying similar functional properties along their length (somatic versus visceral; motor versus sensory; somatosensory, viscerosensory, vestibular, cochlear, etc.). Since all postulated diencephalic columns clearly received specific *retinal input via* the optic tract (some of them several distinct inputs), we soon noted that it was absolutely unclear how viscero/somatic or sensitive/motor sorts of signals were distinguished from visual signals in the columnar diencephalon (i.e., the visual system seemed *anti-columnar*, apart of *entering its visual sensory input through the postulated diencephalic floor and basal plate equivalent region at the hypothalamic chiasma*, a very odd pattern absent in the hindbrain).

As all columnar believers do, we cut our embryonic brains in coronal sections and expected to see distinct histogenetic phenomena to appear at each dorsoventral column, roughly at the superficial sites where visual nuclei were described. Inside any single column, one expected *similar histogenetic phenomena*, perhaps modulated by a gradient, as had been observed in the midbrain optic tectum. While Nissl material possibly allows you to believe that successive coronal sections through a diencephalic column show similar cell types (Kuhlenbeck, 1973), the higher resolutive power of the Golgi-Stensaas method (showing differential details of the neuronal axons and dendrites) immediately refuted this assumption. Unexpected clear-cut boundaries separated distinct intracolumnar fields having different neuron types, crossing obliquely the *theoretically homogeneous* columns, and defining distinct non-columnar blocks of neurons in terms of observable early differentiation sequences. Instead of advancing nicely along a gradient, as the tectal pattern did, the diencephalic Golgi image was a mess of contradictory data we simply could not make meaningful using the Herrick/Kuhlenbeck columnar model. I, thus, started doubting this model and looking around for some alternative model that would explain what we saw.

Colleagues familiar with my subsequent work think that I moved in the 1990s into neuromeres due to evidence coming from gene expression patterns, but this is false. I first learned about neuromeres some 20 years before, but only started to think they might be important for my research due to the problems I had with the interpretation of *Golgi impregnations* in the chick diencephalon in the late 1970s. The genes came up some 15 years later, at which moment I was the expert in neuromeres of the new generation.

During that period, I made another visit to the INSERM U106 in Paris, where I had met previously Jacques Repérant, a researcher of the local Museum of Natural Sciences, who collaborated with Sotelo. He had done his thesis on retinal projections in the pigeon studied with autoradiographic axonal transport methods. We discussed frequently the diencephalon and its visual centers. I told Jacques about my difficulties with the interpretation of developmental Golgi data and the apparent failure of the columnar model to explain the results. He informed me that there existed a long paper (a thesis) of a Norwegian researcher, written in difficult German, and thus hardly mentioned in the literature, which described chicken diencephalic development according to a different, *neuromeric model*. This was the neuromeric thesis work of Rendahl (1924), published in Acta Zoologica, a Swedish journal, whose reference Repérant gave me.

Hyalmar Rendahl was a Norwegian doctoral pupil of Niels Holmgren, histology professor in Stockholm, who also was the tutor of the neuromeric thesis of Bergquist on fish diencephalic development, finished also in 1924, although only published later (Bergquist, 1932). Holmgren is held to be the founder of the rather informal Swedish school of neuromeric students, although he personally never published using this concept, as far as I know. Källén, the later co-author of Bergquist in Bergquist and Källén (1954), whom I met in his retirement during a private weekend visit in 2002, told me that he had never talked with Holmgren personally and that the members of the ‘school’ hardly interacted or met with each other. Even the closer collaborative research relationships of Källén with Bergquist were darkened by the repulse of Källén of the Nazi orientation of Bergquist (they never were friends, as he told me). His former Nazi connection had reduced Bergquist to working after the war as a public school teacher. He was a sort of visiting scientist in Tornblad Embryological institute directed by Källén (a casual conversation of Bergquist with Källén’s father, who was also a public school teacher, led to their meeting). He could use there the comparative collection of embryonic preparations of Ivar Broman, which was publicly available.

The main personal embryological work of Holmgren was on the pallial and subpallial developmental structure of the telencephalon of a series of vertebrates (Holmgren, 1922, 1925). He apparently taught his pupils to examine preferentially *distinct neuronal structures* and the resulting *cytoarchitectonic boundaries* that one may detect in the embryonic *brain wall*. According to him, one should not confide in the delimiting power of *ventricular sulci*, an error-prone procedure widely employed still to this day by the columnar scientists, including originally Herrick and Kuhlenbeck (Källén, personal communication); specific critical comments and demonstrative images about such confusing sulci were shown later in Puelles and Rubenstein (1993). Since I could read German (I studied between age 3 and 15 in an official German school in Tenerife), Jacques Repérant suggested I should obtain the Rendahl (1924) thesis monograph and see whether his neuromeric model somehow helped the interpretation of our diencephalic Golgi data.

I did this as soon as I returned to Cadix, and I was amazed to see the exquisite drawings of neuromeric mantle differentiation and diencephalic interneuromeric boundaries done by Rendahl, which coincided *precisely* with our variously differentiating diencephalic patches of Golgi-impregnated neuronal types (Figure 5). His data predicted all the aberrant oblique boundaries we had found. It turned out that these could be interpreted *against* the opinion of Kuhlenbeck (1973) as *persistent histogenetic landmarks* of the supposedly disappeared early neuromeric units of the diencephalon, thus highlighting solid *Golgi evidence in favor* of the histologic persistence of diencephalic neuromeres. This was later further corroborated by experimental fate mapping of the chick diencephalic neuromeres by my pupil S. Martinez reported in Garcia-Lopez et al. (2004, 2009). The diencephalic thesis of Cristina Zavala, read in the mother institution in Sevilla in tandem with the tectal thesis of Bendala (Bendala, 1978; Zabala, 1978), made extensive use of our neuromeric interpretations inspired in Rendahl (1924); see also in Figure 6 our subsequent comparisons of our chick neuromeric model with the conceptions of Rendahl (1924) and Bergquist and Källén (1954).

However, we hesitated about publishing this conclusion, since I feared that an attack on the solid columnar establishment based on ‘mere’ Golgi data, in the era of experimental neuroanatomic studies and immunocytochemistry, was going to be exceedingly difficult and probably unsuccessful. I started preparing instead a *photographic* documentation of the neuromeric pattern of the developing avian visual centers using Nissl data. I worked for years, even later in Murcia, on these photographic Nissl plates, but they were not published, finally, because I was continually distracted by other preoccupations, such as obtaining tenure (in Cadix) and later two academic jobs I accepted in series in Murcia (vice dean of the medical school and later vicerrector of research), which kept me very occupied up to 1986.

In the meantime, our interest deviated fully into our novel AChE studies of neurogenesis, both in Cadix and Murcia (see below). Part of the neuromeric diencephalic Nissl figures were later included in other studies (e.g., Puelles, 1995). They also underpinned the comparative thesis of my wife Margaret, who examined diencephalic Nissl and AChE adult preparations of chick and several reptiles for consistency with the neuromeric model of Rendahl (she also prepared some frog and urodele brains; Martínez-de-la-Torre, 1985).

In this impasse, I explored widely the literature on brain development in English, German, French, Italian, and Spanish, to learn as much as possible about neuromeric models and check results recorded in columnar studies that possibly admitted a neuromeric reinterpretation. There were many examples of that sort. I was surprised to find that neuromeric reports actually preceded historically the proposal of the columnar model of Herrick (1910; see, for instance, Orr, 1887; McClure, 1890; Locy, 1894, 1895; Neal, 1898; Hill, 1899; Von Kupffer, 1906; Ziehen, 1906). I tried to understand why sound neuromeric ideas, as I now held them to be, had been left aside by morphologists in favor of functionally tendentious and ultimately morphologically wrong columnar ideas, to the point of neuromery disappearing from the textbooks altogether. The publications of the Nordic school were not easily found, being hidden in Swedish journals not available in many European or American libraries, but the main references were reported by Vaage (1969), as well as by Kuhlenbeck (1973). Luckily, Carlos Maynar, an old friend from my German-school days in Tenerife lives in Stockholm, and he obtained for me photocopies of all relevant Swedish documents at the Karolinska Institute (all the works by Holmgren, Rendahl, Bergquist, Källén, and Söderberg, among others).

Careful analysis of these papers suggested several problems: (1) *Well-fixed* specimens were mixed in the descriptions with *badly fixed* ones (Bergquist and Källén largely studied in their collaborations embryonic material prepared one generation before by Ivar Broman). (2) The apparent fading of the early interneuromeric constrictions was not properly investigated. (3) Bergquist and Källén (in 1954 and elsewhere) came to believe, probably due to the variable quality of their material, that neuromeric bulges were quite evanescent and appeared and disappeared sequentially in three temporal series (distinguished as proneuromeres, neuromeres, and transversal bands); they thus emphasized unstable patterns of bulges, which unfortunately helped the idea that neuromeric phenomena were intrinsically variable and transient, and thus possibly irrelevant. Modern analysis of neuromeres by fate mapping or experimental transgenic progeny analysis has revealed that the primary interneuromeric constrictions systematically persist as hidden *molecular boundaries* in the adult brain (see Watson et al., 2017a,b, 2019). (4) Early students of neuromeres did not attempt to investigate their possible modular functional properties in the adult brain. These aspects are increasingly being studied today (neuromeric analysis of modular aspects of serotonergic raphe nuclei, respiration centers, somesthetic, vestibular and cochlear function, reticular formation, visual forebrain subsystems, etc.).

In contrast, at the turn of the 20th century, notable progress had been made with the functional columnar classification of fiber components of the cranial and spinal nerves. This led to the *columnar theory* for the hindbrain of Gaskell (1889); Johnston (1902), and Herrick (1903). Herrick (1910) extrapolated conjecturally such initial brainstem functional columns into the forebrain, thus formulating his *columnar forebrain model* (Figure 4B). In it, he arbitrarily changed the earlier sound concept of His of the forebrain axis (His, 1893, 1904; Figure 4A), disregarding in so doing the clearcut contradictory morphologic evidence offered by the cephalic flexure in all vertebrates, indicating that the axis does not end in the telencephalon (Figures 4A–C). It was Herrick’s promise of *functional explanations* in terms of ‘somatic’ and ‘visceral’ or ‘motor’ versus ‘sensory’ columnar specialization, jointly with the apparent complexity and intrinsic variability of neuromeric phenomena presented as devoid of function, what led the field to abandon massively the ‘merely transient’ neuromeric phenomena. Neuromeric studies were also held to delve fruitlessly (from a functional viewpoint) on idealistic ‘formanalytic notions’ reeking of the discredited German *Naturphilosophie*. Herrick (1933, 1948) actually disdained the substantial embryological helping hand of Kuhlenbeck because the latter employed a ‘formanalytic’ approach based on Herrick’s own sulci. Herrick claimed that brain morphology had to be strongly *guided* by functional analysis (i.e., connections), an idea famously defended before by Cuvier in a wider context. We have developed modernly the contrary viewpoint, namely, that functional explanation needs to be *preceded* by sound morphologic and developmental analysis (see last chapter in Nieuwenhuys and Puelles, 2016).

Remarkably, the modern neurobiological field, which persists unwittingly on columnar morphologic theses, has *silently* ceased to assume structural and functional *homogeneity* of the forebrain columns. It now contradictorily admits, in general, as is described without commentary, that the formerly ‘uniform’ ‘columnar diencephalic units’ are now bristling with nuclear *parts* that *do different things* (think of the hypothalamus, thalamus, prethalamus, pretectum, even the epithalamic habenula). Persistent columnar followers such as Swanson (2012, 2018) do not attempt to *explain* how the functional and structural complexities that appear in their investigations emerge ontogenetically out of the postulated columns in all vertebrates, and still center their attention instead on the connectivity and circuit functions apparently performed by dedicated parts of the columns. There is to this day no columnar theory explaining how an embryonic *column* diversifies into a collection of *distinct nuclei*, either in the hypothalamus, the diencephalon, the midbrain, or the hindbrain. In contrast, the prosomeric model has incorporated dorsoventral and anteroposterior patterning and regionalization effects leading to molecular definition of the component progenitor areas that produce specific cell types or nuclei. The columnar model is incompatible with these experimentally demonstrated patterning effects because its arbitrary length axis ending in the telencephalon prohibits it (the implicit meanings of the columnar descriptive terms *dorsal*, *ventral*, *anterior*, and *posterior* are inconsistent with what we now know of brain patterning).

The speculative columnar structure of the forebrain has nevertheless become dogmatically established in the literature after a century as a conventional *truth* or *fact*. No expert acknowledges that there is an underlying theoretically fallible *columnar model* dating from 1910, with a number of now very doubtful, when not clearly false, assumptions, that is responsible of a major scientific impasse. Criticisms of the columnar model of Herrick are labeled as unjust attacks on a ‘straw man’ (this is personal experience), since modern neurobiologists no longer are conscious of following the columnar model. This *factual character* of the columnar model in practice was the barrier I had in front once I realized the obsolescence of this model and the need to substitute in its place a neuromeric brain model corrected from earlier errors *throughout neuroscience*. I needed a modern technique which could be used to reverse the *status quo*. In retrospect, what I obviously really needed were *gene expression patterns* and the *experimental analysis of neural patterning*, but we only got such data in the 1990s. These new research instruments could not even be imagined in the late 1970s, particularly not in Cadix or Murcia, where no molecular research was performed by anatomists; I was literally thought to be out of my mind when I expressed to colleagues interest in molecular genetics for progress in embryology and anatomy.

What serendipitously seemed to suggest a solution for this hard problem was a collateral line of study I started in Cadix, after reading an introductory book on histochemistry. This was the employ of simple acetylcholinesterase (AChE) histochemistry, which I first wanted to apply to the old issue of the oculomotor migration across the midline. Since these were motoneurons and supposedly cholinergic cells, known to be cholinesterase-positive in the adult, perhaps they could be selectively traced histochemically in embryonic wholemounts, leaving any surrounding elements unstained. I thus did wholemount reactions with the one-solution AChE procedure of Karnovsky and Roots (1964) on the fixed heads of young chick embryos and later cleared them for visualization. A division of the heads through the midline helped the mapping of the stained neurons through the unstained ventricular zone in the translucent head halves. Soon I perfected the method by dissecting after the reaction the skin and meninges away under an operating microscope using sharp tungsten needles.

The big surprise obtained already from the first specimen treated was that the AChE staining was not restricted to presumptive cholinergic motoneurons but characterized apparently *all postmitotic and differentiating young neurons* present in the brain. Neuroepithelial progenitor cells instead remained conveniently negative, with few exceptions at sites where radial glia palisades develop (e.g., epichordal floorplate and the diencephalic zona limitans). A quick check of the histochemical literature on AChE showed that this result on general early expression of neuronal AChE had been extensively reported and analyzed already since the 1950s, although nobody had used wholemounts to study the overall patterns (see specific references to this literature and comparisons with other early neuronal markers in Puelles et al., 1987a). Frozen or cryostat sections habitually used for histochemistry did not allow an overview, unless graphic reconstruction was attempted, a method hampered by the usually incomplete cryostat section series.

Embryos fixed at progressively different stages and processed according to our whole mount protocol showed remarkably a neurogenetic progression which did not agree with the neurogenesis pattern then suggested in the literature, namely, a hypothetical general wave of neurogenesis expanding rostrally and caudally from an early starting spot in the medullary brainstem. Instead, we systematically observed discontinuous cell groups, which appeared heterotopically and heterochronically, with clear-cut orthogonal transverse and longitudinal boundaries (see Figure 6B). I soon realized that the discontinuities in neurogenetic progress seen both in the forebrain and the hindbrain were consistent with the diencephalic neuromeric models of Rendahl (1924) and Bergquist and Källén (1954) analogous models of the rhombomeres (e.g., Vaage, 1969, 1973; Figures 6A–C; check for review Amat et al., 2021). Our study of AChE wholemounts accordingly increasingly concentrated most of my efforts during the last period in Cadix (1978, 1979 and first half of 1980), in which endeavor I was helped considerably by a new collaborator and thesis pupil, J. A. Amat (see below). The same study still continued for several years more in Murcia before we first attempted publication in 1986.

My wife and I started on the side in Cadix a neuromeric Golgi study of the developing isthmic nuclei (the theme was inspired by the extraordinary Nissl neuromeric analysis of that domain by Vaage, 1973). We complemented these data some years later in Murcia with autoradiographic results showing differential neurogenetic timing in the relevant neuromeric units (Puelles and Martínez-de-la-Torre, 1987). Isthmic nuclei were classically ascribed to the midbrain, but they are instead found spread across two to three neuromeres between the midbrain and the rostral hindbrain, as was first cytoarchitectonically demonstrated by Vaage (1973) and corroborated by us. We later also added differential gene expression patterns (Aroca and Puelles, 2005; Hidalgo-Sánchez et al., 2005; Aroca et al., 2006; Puelles et al., 2012b; Watson et al., 2017c). The Golgi preparations also illustrated the existence of an isthmic tangential migration that translocates the primordial subpial hindbrain isthmic cell plate rostralwards. Hidalgo-Sánchez et al. (2005) reported that the midbrain portion of the isthmic complex—the so-called magnocellular isthmic nucleus—originates specifically within the *second* midbrain mesomere (m2), previously described classically, but wrongly considered by Palmgren (1921), Vaage (1973), and us in Puelles and Martínez-de-la-Torre (1987) to be an oddly *atrophic* thin segment devoid of neuronal derivatives. We recuperated the ‘normal’ m2 mesomere concept once we discovered there exist specific and molecularly distinct m2 neuronal derivatives (Hidalgo-Sánchez et al., 2005; Puelles et al., 2012b,^2007^, ^2019^; see also my reference atlases for the Allen Developing Mouse Brain Atlas). We proposed the introduction in the neuroanatomic terminology of the term *pre-isthmus*, alluding to this novel midbrain anatomic domain just *in front* of the isthmus proper and *caudal* to the inferior colliculus.

## Academic Tenure and Murcia Work Up to the Nineties

I won in 1979 *via* competition in Madrid a tenured *adjoint neuroanatomy professor* position in the university of Murcia. This was later transformed by law in a standard *full professorship* in 1983. I finished the 1979–1980 term in Cadix. In the summer vacances of 1980, Margaret and I did a stay at the Göttingen Max Planck Institut für biophysikalische Chemie, Neurobiologie Abteilung, visiting the laboratory of Günter Rager. There we learned techniques for HRP axonal transport and electron microscopic embedding of Golgi preparations. In Göttingen, I studied HRP-labeled retinal projections on chicken, which I mapped satisfactorily according to the neuromeric model, but which unfortunately remained largely unpublished. We reported only some details about the topography of the blind retinal papilla representation upon the tectum and several diencephalic visual centers. The data were of use in any case, since they were reflected in our subsequent neuromeric chick brain atlas (Puelles et al., 2007).

At the beginning of the 1980–1981 term (September), we arrived in Murcia, in whose medical school and anatomy department Margaret and I worked thereafter for 40 years. The laboratory had to be organized from zero, though. No silver or Golgi method worked because double distilled water contained too much organic material, so that silver and chromium salts systematically precipitated. This was the reason why I ceased to use those techniques and concentrated on the AChE material. We had to install a system for de-ionized water, which took some time and the arrival of national funding.

We first obtained in 1981 a grant from the ministerial central funding agency in Madrid (I had not applied before, I do not know exactly why: perhaps no public grants were available during my previous formative years or at least they were not known to me; Génis-Gálvez had been funded habitually by a private foundation). Thereafter, we continued having sufficient national grant support on a 3-year renovation basis up to my retirement in 2018 (the last 5–6 years we had an extremely hard to get Excellency grant—only two were given per year in Spain). We also had extra help in parallel from several European projects, a Human Frontiers grant, and a NIH project. In recent years, we also obtained some significant funding from the local regional government in Murcia (Séneca Foundation). All this funding helped us to expand and modernize the lab to international standards, and we established infrastructure and know-how for molecular biology procedures, as well as automatized scanning of our slides.

In our first project, we studied neurogenetic patterns in the chick brain using thymidine autoradiography. This accompanied our whole-mount AChE visualization of differentiating young neurons. Parts of the autoradiographic data on the isthmus, midbrain tectum, and diencephalon (thalamus) were published. Others on oculomotor nuclei, hindbrain, diencephalon, and cerebellum remained unpublished.

The second project approached detailed comparison of diencephalic neuromeric development between chick and lizard embryos (*Gallotia galloti*; the lizard eggs were obtained by colleagues in our home country, Tenerife, where this species is endemic). We also checked diencephalic neuromeric structure in a variety of amphibians and other reptiles in comparison with the chick. This project was accompanied by HRP experiments studying visual projections in the chick, the turtle, and the rabbit (none of them published). The doctoral theses of my wife (Martínez-de-la-Torre, 1985) and of my first Murcia pupil, Salvador Martínez (Martínez, 1987), emerged out of this project.

Later we introduced immunoreactions, largely of calcium-binding proteins and cadherins (collaboration with Ch. Redies in Germany), and continued collaborating with Carmen M^a^ Trujillo in Tenerife. This resulted in the theses of Loreta Medina and Carmen Diaz, defended in 1990 at the University of La Laguna in Tenerife, with various publications in 1991. Both subsequently did postdoctoral work in my laboratory before going abroad and thereafter returned in one way or another for additional time to my laboratory. We still continue in contact and occasionally publish together. We also collaborated over the years in several papers on the lizard brain with colleagues led by Salvador Guirado in Málaga, on the lamprey brain with M. A. Pombal in Vigo (Pombal and Puelles, 1999; Pombal et al., 2009; Martínez-de-la-Torre et al., 2011), on the frog brain with A. González in Madrid (as well as work by my thesis students Aurora Brox and F. Javier Milán), and on the zebrafish brain with M. Wullimann (Wullimann and Puelles, 1999; Wulliman et al., 1999). More recently, we even had a go at neuromeres in the cephalochordate Amphioxus forebrain and hindbrain (Albuixech-Crespo et al., 2017), where an incipient but remarkably incomplete brain Bauplan was found.

Our comparative rationale was that, if the neuromeric model was any good, it had to be capable of application in all vertebrates, and its early forms might be already detectable in cephalochordates (this was indeed what we found in the cited Amphioxus paper, with unsuspected novel collateral aspects). Throughout these years, we worked much (demonstrating the wide usefulness of the prosomeric model in such studies, as noted also by other laboratories that incorporated to the quest) and obtained large amounts of histological material of various types, kept in our histological collection. It was all very useful for advancing my evolutionary ideas, although we did not publish large parts of it since we first had to introduce the neuromeric model into the field (we only achieved this by publications in 1987 and 1993), and then we had to lift our lab to the capacity to perform molecular mapping studies.

The first published gene pattern I noticed that showed a neuromeric pattern appeared in the report of Gaunt et al. (1986) on Hox1.5 expression in mouse embryonic hindbrain. The authors showed that Hox1.5 expression had a distinct rostral limit at a constriction of the hindbrain neural wall. They apparently did not realize (as I immediately did) that this constriction was an interrhombomeric boundary. I noted the enormous relevance of that sort of data for our project, since all interneuromeric and intraneuromeric boundaries now probably could be explained and visualized as patterned gene expressions. I visited the laboratories of friends in Paris (Marion Wassef at L’ École Normale) and Madrid (Angela Nieto at the Cajal Institute) in order to learn personally *in situ* hybridization procedures and associated molecular concepts and techniques.

Jose A. Amat, who originally had been one of my neuroanatomy students in Sevilla, moved to Cadix to work with me when he finished his medical studies. He later followed us also to Murcia, where he read his thesis using wholemount AChE for the study of neuromeric patterned neurogenesis (Amat, 1986). Our main neuromeric publication of this period included the AChE data for chick midbrain, diencephalon, and hypothalamus (Puelles et al., 1987a). Another part of our joint AChE work in Cadix and early years in Murcia, including hindbrain data that were reported in the thesis of Amat remained unpublished many years due to circumstances associated with his move to the States. It was prepared recently for publication after we reestablished contact across the Atlantic in 2019 (Amat et al., 2021).

Our histochemical results on the dissected wholemounts of developing chick embryo brains represented an independent modern test of the neuromeric model, using impeccably fixed and closely staged specimens. Moreover, our approach using transparency of the wholemounts provided material that evaded the difficulties due to the apparent loss of limit constrictions or a dependency on section planes, and we did not have sectioning and reconstructing complications, as did other studies using cryostat sections (Figure 6B). Moreover, we looked preferentially at *intrinsic patterns of neurons* inside the brain wall, as was recommended originally by Holmgren. As a research project, it represented altogether a stronger basis for our attempt to attack the columnar establishment with our revamped neuromeric model. Serendipitously, it also prepared us conceptually, and trained us practically, for the forthcoming morphologic interpretation of relevant gene expression data in the early 1990s.

A parallel paper entirely done in Murcia (Puelles et al., 1987b) was an experimental fate mapping study on the rostral end of the rostral neuropore. A small piece of black Nylon thread was inserted at the rostral end of the rostral neuropore at successive stages of neurulation. The neuropore was allowed to close, and then the specimens were fixed and stained whole-mount with AChE. We examined the cleared preparations for the final midline position of the black threads, checking whether this position changed from early to late interventions (as postulated by several experts), or was always at a fixed point (as proposed by Von Kupffer, 1906). The second option won. The observed pattern of closure of the rostral neuropore, thus, established experimentally for the first time the rostral end of the neural roofplate as corresponding invariably to the prospective site of the anterior commissure in the septal commissural plate (this point was subsequently further corroborated by other experiments; see below). It was a partial attempt to produce a more precise definition of forebrain longitudinal zones; this was essential for pitfall-free definition of transverse neuromeres orthogonal to them. We later did a second analysis of this point with quail-chick grafts—Cobos et al. (2001)—whose results confirmed fully our previous conclusion. Similar data were independently obtained for the mouse by Inoue et al. (2000) and for amphibians by Eagleson (see review in Rubenstein et al., 1998). Defining similarly with precision the rostral end of the floorplate needed gene marker results that were only obtained some years later (thesis of Bardet, 2007; Puelles et al., 2012a; Puelles and Rubenstein, 2015). We also marveled at the discovery of *Nkx2.2* as a gene marker for the forebrain alar-basal boundary (Shimamura et al., 1995), another essential longitudinal landmark (see also Puelles et al., 2012b,c; Puelles and Rubenstein, 2015).

The Puelles et al. (1987a) manuscript on AChE data in a neuromeric pattern was first submitted in 1986 after first showing it to Sotelo in Paris, who passed it on with a favorable comment to S. Palay, the editor-in-chief of the Journal of Comparative Neurology. The paper evaded criticizing the columnar model; it just showed abundant photographs of what we had found and stated that the patterns were clearly consistent with the neuromeric models of the past. Palay sent back reviews suggesting that perhaps because we worked in a Spanish provincial city we were not aware that this subject of neuromeres had been discarded many years before and that we should examine modern literature in university libraries in Madrid or Barcelona. The rather supercilious reviewers apparently thought that we somehow were using ideas and literature citations left behind by our grandfathers. Palay nevertheless asked us to send back a shortened and appropriately modified version of the text.

In my response letter, I highlighted the point that none of the reviewers had found anything to criticize or reinterpret about our photographic data, the main evidence we were offering, so that we did not see in which sense we should change our interpretation, which seemed to us the only possible one. Our results seemed indeed to revitalize old forgotten neuromeric notions, but the age of such notions and their conventionally disregarded status were not our responsibility. We just stood on a novel sort of evidence—unobjectionable wholemount AChE reactions—that strongly agreed with these old ideas, obviously disagreeing with the contrary columnar ideas *a la mode*. Moreover, I made known to Palay the vast preliminary analysis of relevant literature I had performed over the previous 10 years (including not only everything in English but also in other important scientific languages). I included a synopsis of the journal and book material obtained from the libraries of the Karolinska Institute in Stockholm, the Max Planck Institute in Göttingen, and the INSERM U106 in Paris. We did shorten the text a bit, and the second version was accepted without any further discussion. I think that Palay treated us very fairly, better than I expected, and he probably acted against the opinion of the reviewers, in agreement with the important supporting position of Sotelo. Overall, in our first decade working in Murcia (1981 to 1991), we published 15 papers, three of them dealing with neuromeric subjects.

## Meeting John L. R. Rubenstein and Birth of the Prosomeric Model

In the summer of 1992, Margaret and I traveled to La Jolla (California) to work 1 month on fish and amphibian brains with the comparative neuroanatomist Glenn R. Northcutt at the Scripps Institute of Oceanography. The project was to show him in practice, using his own material (previously unknown to us), evidence in favor of the neuromeric brain model.

We first tried some *Ambystoma tigrinum* (salamander) brain specimens cut sagittally, which we immunoreacted with calretinin (note that *Ambystoma* was the amphibian species most studied by Herrick in his columnar studies and also the object of his recapitulative book “The Brain of the Tiger Salamander,” Herrick, 1948). I thought that the calretinin—CR—marker (used before by us in chick and lizard embryos) might be favorable for identifying a positive *dorsal thalamus* against the CR-negative ventral thalamus and pretectum domains (these represent the three diencephalic neuromeres in the alar plate). Such differential staining should also show that the corresponding limits are *transversal* (i.e., contrary to columnar expectations postulating at least one of them—the interthalamic zona limitans limit—to be *longitudinal*). The result was spectacularly positive.

In this pedomorphic amphibian, most diencephalic neurons differentiate close to the ventricle (retaining an embryonic non-migratory state) and with routine stains there are hardly any cytoarchitectonic differences or limits from one diencephalic part to another; the mantle layer looks like a continuous periventricular sheet. That is probably the reason why Herrick was led to use ventricular sulci as regional boundaries. Northcutt, thus, had knowingly chosen this material to test our capacity to identify any neuromeric boundary therein. He was, thus, amazed to see the clearly transverse limits of the thalamus, outlined by the contextual landmarks of the obviously transverse retroflex tract and the orthogonally running longitudinal dorsal (thalamic) and ventral (tegmental) roots of the cerebral peduncle; all of them also are calretinin-positive, likewise as the longitudinal optic tracts (Figure 4C).

During our stay in La Jolla, the yearly meeting of the American Society for Neuroscience was held nearby in San Diego, and I attended it as an observer (this was my first time at the SfN). I heard an oral intervention by a young researcher from San Francisco—John R. L. Rubenstein—who presented the diencephalic and telencephalic expression pattern of a newly discovered gene then called *Tess1* (for his daughter Tess; this was his first output in gene mapping). This gene subsequently was reclassified as Dlx2 (a member of the important Dlx family, functionally involved in the generation of forebrain GABAergic neurons, among other properties; *distalless* refers to lack of terminal leg parts in Dlx-mutant Drosophila). Rubenstein presented at the SfN a *columnar* interpretation of the diencephalic expression pattern of Dlx2, which seemed to me problematic in several aspects. I thought that a more significant interpretation was possible using the *neuromeric* model.

I approached Rubenstein afterward outside the room, introduced myself, and told him about this possibility of an alternative interpretation. He answered that it did not surprise him because all the (columnar) American neuroembryologists he had approached for help had told him that the pattern he had discovered was strictly meaningless. As a molecular biologist, he did not believe that a gene pattern in the developing brain could be meaningless, so he was looking around for alternative ways to find its meaning. In fact, he opened his briefcase and showed me a reprint of our AChE paper of 1987 in the chick (Puelles et al., 1987a). Somebody he had recently consulted had given it to him as a possibility to explore alternative interpretations.

Rubenstein invited me to visit his lab in San Francisco the next weekend, in order to discuss further the issue, my ideas, and the possibility of collaboration. There was the difficulty of our separation by the whole continent and the Atlantic; note that neither email nor internet were in use yet in 1992, so that communications were limited to the post and long-distance phone calls. I flew to San Francisco, and John got more and more interested in the model I was using. Although I had perfected the model of Rendahl (1924) mainly for the avian and reptilian brains, he needed its application to mouse embryos. I assured him that this posed no difficulties.

**FIGURE 7:**
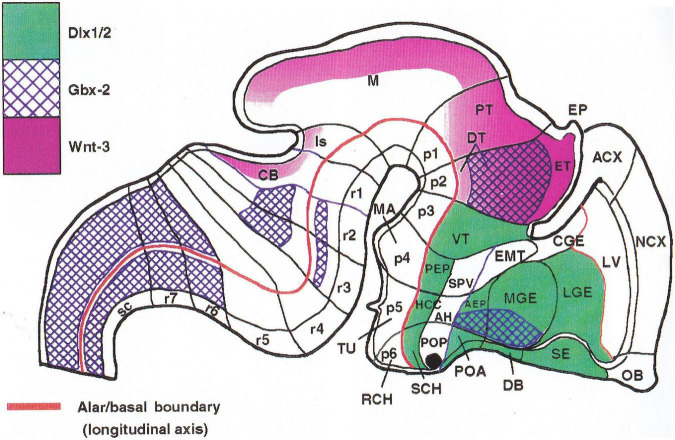
First version of the prosomeric model, from Bulfone et al. (1993). The genes mapped are color-coded. Note that, at variance with the bipartite chick hypothalamus of Puelles et al. (1987a; see Figure 6B4), we postulated three hypothalamic prosomeres p4–p6 for the mouse. This was an error that we corrected subsequently.

In the end, John decided that we could try to collaborate at a distance, in the following way. His lab would prepare the mouse *in situs*, following my instructions on sectioning planes. He would send me periodically photographic positives through the post (large packets of them), and I would send him drawings and written reports of my interpretations, commenting on the developmental and morphologic *meanings* that could be extracted from such material. Every summer in the near future I would spend August (my vacances) in San Francisco, so that we could discuss things in person. We did this roughly up to 2008, when I started working on the Allen Developing Mouse Brain Project in Seattle (it was John as head of the organizing committee who posted me there). At the beginning, John called me occasionally up on the phone at odd hours to discuss my posted comments. Roughly after 1 year, we were able to write our first papers reporting a number of gene patterns that were topologically consistent with a ‘segmental’ or neuromeric interpretation, that is, we produced the ‘*prosomeric model*,’ as it was soon named (Figure 7; Bulfone et al., 1993, 1995; Puelles and Rubenstein, 1993; Rubenstein et al., 1994, 1998; Puelles, 1995; Shimamura et al., 1995, 1997).

**FIGURE 8:**
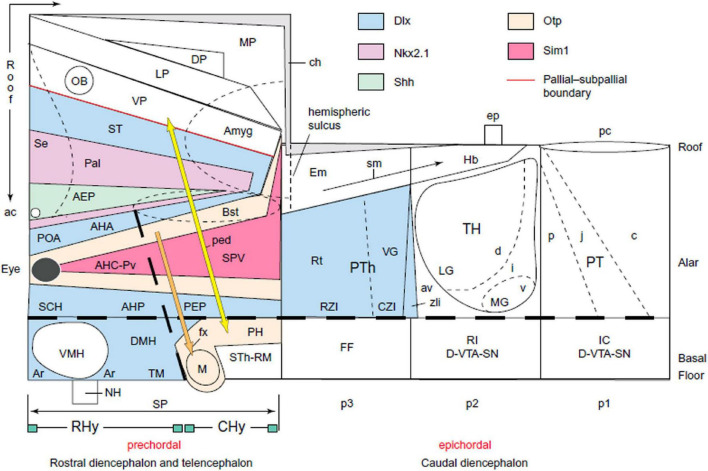
Schema representing the prosomeric model as actualized in Puelles and Rubenstein (2003). Note that the secondary prosencephalon (SP), comprising hypothalamus and dorsal telencephalon, was left devoid of any prosomeric subdivisions. We knew that our former three units were incorrect, but had not yet found the solution for the neuromeric connection between the hypothalamus and the telencephalon. Note that at this stage we still believed in the ascription of the SP to a prechordal region of the brain. This was part of the problem and was resolved subsequently jointly with the hypothalamus issue (see Figure 9). We also used in this schema the concepts ‘rostral’ and ‘caudal’ diencephalon, which were later suppressed to avoid confusion with columnar notions (it is preferred to wholly separate the hypothalamus concept from the diencephalon proper). The three alar diencephalic domains show tentative schematic subdivision patterns that were largely corroborated subsequently, particularly the rostrocaudally tripartite pretectum (PT; Ferran et al., 2007), and the dorsoventrally tiered thalamus structure (d, i, v; Th; Redies et al., 2000; Puelles and Martinez, 2013). The prethalamus (PTh) was recently found to be rostrocaudally tripartite (Puelles et al., 2020). The inclusion of the prethalamic eminence in the prethalamus (Em), just rostral to the thalamic habenular area (Hb), was a change introduced in this schema. The hypothalamo-amygdalar bipartite spike was then based on Fan et al. (1996) and has been confirmed recently as the ‘hypothalamo-amygdalar corridor’ in Garcia-Calero et al. (2021).

**FIGURE 9:**
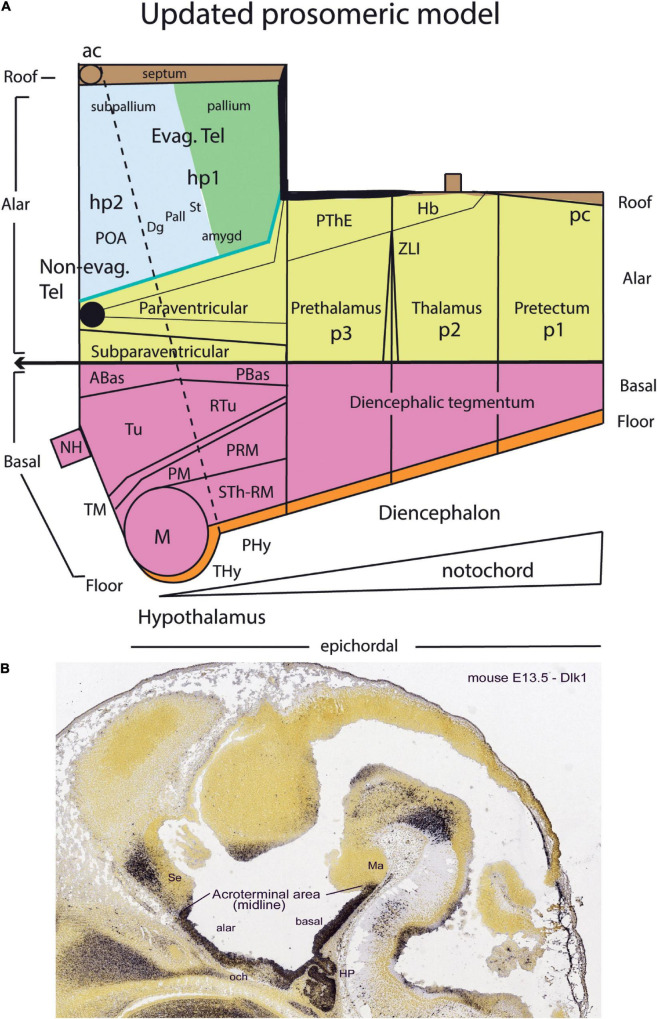
Updated prosomeric model of Puelles et al. (2012a) and Puelles and Rubenstein (2015) **(A)** and acroterminal domain **(B)**. **(A)** In this schema, which resolves the uncertainties we had before, the whole forebrain is now held to be epichordal, as a result of recognizing that the notochordal tip reaches the mamillary pouch, thus inducing there the rostral end of the floor plate (a hypothalamic floor plate). This means as a big conceptual change that there is nothing more rostral than the mamillary pouch (M), although the preoptic area (POA) is equally rostral, as are all acroterminal entities (see also Puelles, 2018). This allows the definition of two complete hypothalamo-telencephalic prosomeres, called hp1 and hp2, which reach independently the commissural roof plate. Note they are organized dorsoventrally into floor, basal, alar, and roof longitudinal domains, like all other neuromeres. The alar plate regions are divided between alar hypothalamus and telencephalon (the later is evaginated in hp1, but represents the non-evaginated preoptic area in hp2; POA). The adjustment of the floor domains pushes the tuberal region (Tu) with the neurohypophysis (NH) out of the floor and into the rostral median end of the brain, the basal part of the acroterminal domain (see **B**). **(B)** Sagittal midsagittal section through an E13.5 mouse embryo, labeled with a Dlk1 riboprobe that identifies selectively the acroterminal area of the rostral hypothalamo-telencephalic prosomere. Note this rostromedian domain encompasses alar and basal portions, reflecting its participation in standard dorsoventral patterning due to antagonistic floor ventralizing and roof dorsalizing morphogens (note also alar and basal hypothalamic partitions reflecting dorsoventral patterning). The acroterminal area also receives strong signals from the prechordal plate, whose cells migrate ventrodorsally in front of it. The prechordal tissue is held not to form ‘rostral’ to the notochord, but ‘dorsal’ to its rostral end beneath the floor plate, and extends progressively dorsalward toward the roof plate (see Puelles, 2017; Diaz and Puelles, 2020).

**FIGURE 10:**
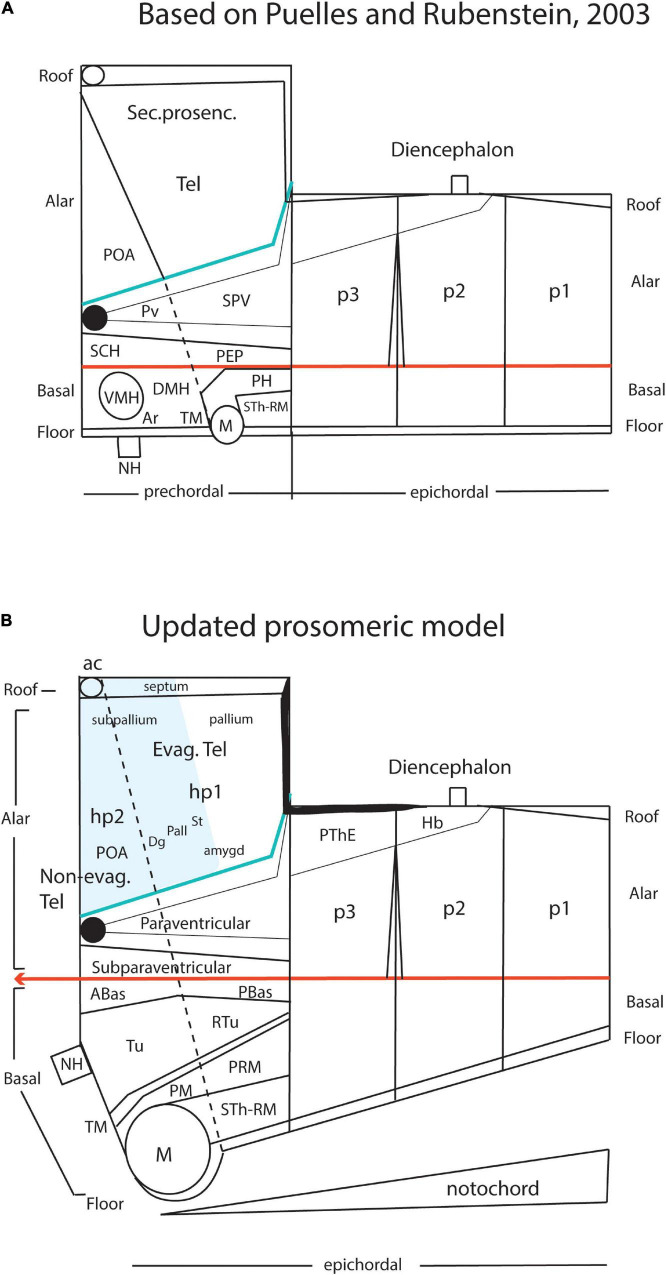
Comparison of the prosomeric models conceived by Puelles and Rubenstein (2003) **(A)** and Puelles et al. (2012a) **(B)** to visualize the significant changes introduced in the updated version, which were explained in detail in Puelles and Rubenstein (2015).

For a number of years, we published two to three joint papers per year, some of them being collaborations with colleagues that became interested in the neuromeric interpretations we offered with our model. Collaborations between John and myself often involved some of our collaborators (several of mine did stays in John’s laboratory). They have continued up to the present at a slower pace, with a total of 42 published reports. Several more are still in course. The initial forebrain prosomeric model was examined critically in Bulfone et al. (1995) and Puelles and Rubenstein (2003; Figure 8), identifying some difficulties that we continued to analyze. The model was eventually significantly updated based on new ideas developed in Murcia during the thesis of Bardet (2007) on the developing chick hypothalamus, leading to a massive analysis of the mouse hypothalamus reported in 2012 (Puelles et al., 2012a; Figures 9A,B). The more recent Puelles and Rubenstein (2015) and Puelles (2018) publications explained in detail these changes, particularly as regards the hypothalamus and its relations with the telencephalon, understood as a hypothalamic dorsal evagination (Figure 10). In Puelles et al. (2012a), we introduced also the important novel concept of the *acroterminal domain*, representing a topologically transversal linear rostral end of the neural tube, whose structure relates to unique prechordal plate patterning effects (Figure 9B). These updates provided a stronger *causal basis* for the different parts of the model in terms of early AP and DV patterning, increasing its overall coherence and explanatory power (Puelles, 2017, 2018; Puelles et al., 2019).

**FIGURE 11:**
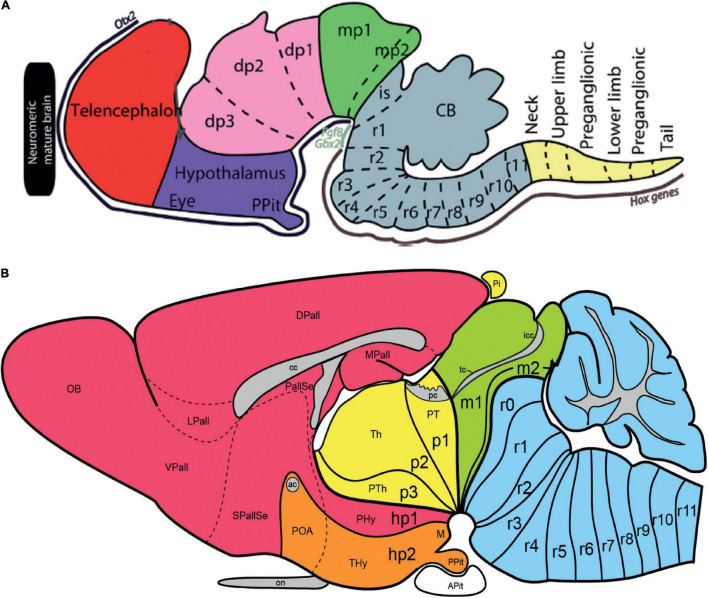
Schemata illustrating embryonic **(A)** and adult **(B)** location of rhombomeres within the prosomeric model. **(A)** Updated schema taken from Watson et al. (2017b), in which the forebrain prosomeric fields (including the secondary prosencephalon [red/blue], diencephalon [pink], and midbrain [green]) appear under the control of the Otx2 transcription factor. In contrast, prepontine rhombomeres r0/is and r1 relate to the area of influence of the secreted Fgf8 morphogene and the Gbx2 transcription factor, while the pontine (r2–r4), retropontine (r5,r6), and medullary (r7–r11) hindbrain rhombomeres plus the spinal cord obey to differential Hox gene signals. These markers can be used to identify the different neuromeres in the mature brain (Tomás-Roca et al., 2016; Watson et al., 2017a,c). **(B)** Schema representing the whole set of prosomeric units in the adult brain (the cerebellum belongs to r0—the vermis—and r1—the hemispheres). Note the large evaginated telencephalic development corresponding to hp1 (red), while the rostral end of the brain corresponds to the acroterminal domain within hp2 (orange). The bipartite hypothalamus is divided into peduncular hypothalamus (PHy) within hp1 and terminal hypothalamus (THy) within hp2. The axis of the brain clearly bends ventrally at the cephalic flexure, where a number of interneuromeric boundaries converge pialwards, and also shows a less marked dorsal bending at pontine levels, causing there also some convergence of neuromeric boundaries at the ventricular surface.

Meanwhile, parallel work we did in Murcia expanded the model of *hindbrain rhombomeres* and *midbrain mesomeres* (Figure 11; Marín and Puelles, 1995; Cambronero and Puelles, 2000; Aroca and Puelles, 2005; Hidalgo-Sánchez et al., 2005; Aroca et al., 2006; Marín et al., 2008; Puelles, 2013; Puelles et al., 2013; Tomás-Roca et al., 2016). Our analysis combined hindbrain fate mapping at both the overtly segmented (Marín and Puelles, 1994) and apparently unsegmented parts of the hindbrain (Cambronero and Puelles, 2000) with mapping of additional families of Hox genes, or other genes (e.g., Fgf8; Marín et al., 2008 in the chick; Di Bonito et al., 2013, 2017; Tomás-Roca et al., 2016; Watson et al., 2017c in the mouse). These markers had remained unmapped in rostral and caudal hindbrain regions by earlier students of rhombomeres due to the previous arbitrarily assumed restriction of true neuromery to r2-r6. We, thus, identified the existence of *cryptorhombomeres* (r0, r1; r7–r11; Figures 11A,B), that is, *hidden rhombomere units* that early on seem overtly undelimited one from another (no visible early constrictions) but display nevertheless distinct molecular Hox gene limits coinciding antimerically with adjacent somites. These cryptorhombomeres were further shown to participate in the formation of plurisegmental (modular) hindbrain nuclei and sensory columns in a manner entirely comparable to the overtly delimited rhombomeres (Cambronero and Puelles, 2000). We, thus, count a total of 12 hindbrain rhombomeres (r0-r11; r0 being the isthmus); r0 conceived as a separate unit was verified using the labeled progeny of *Fgf8*-positive progenitors (Watson et al., 2017a). Equally important for the progress of the prosomeric model were the experimental embryologic contributions of my pupil Salvador Martínez on the analysis of the isthmic and mid-diencephalic secondary organizers, as well as on clone-isolating properties of interrhombomeric and forebrain limits.

## Conclusion

The prosomeric model has proved to present many possibilities, both in our hands and increasingly in those of others the world over. Having it in mind increases the scientist’s awareness of landmarks signifying specific brain parts and their boundaries in much more detail than was possible with the old columnar model. Morphological interpretations, thus, become richer and more meaningful. When examining new materials, such as the brain of previously non-studied species, the prosomeric model predicts, on the basis of minimal characteristic observations, specific details that are likely to be observed or expected (their general position and even their possible relationships with other details). Less understood parts of the brain stand out (become salient) when seen through the viewpoint of the model, and the spatial and causal assumptions of the model readily suggest old or new ideas that can be applied or examined in such places. The design and interpretation of new experiments and their results are clarified and aided, as well as the reinterpretation of previous results existing in the literature. The model has been scarcely developed yet in functional directions (but see, e.g., Cisek and Kalaska, 2010; Cisek, 2019, apart what was already mentioned in the text, mainly on the hindbrain neuromeres e.g., work of J. Champagnat, H. Straka, R. Baker, E. Gilland, D. Noden), but I believe there are many possibilities implicit in the modular serial arrangement of neuromeric components as well as in the dorsoventral microzonal structural aspects. In fact, I expect a revolution in neurophysiology and a step forward in cognitive studies. As a whole, the model has been already extraordinarily successful in the fields of developmental neurobiology (including particularly fate-mapping and patterning studies) and comparative *evo-devo* studies. It is difficult at the present stage to envisage what sort of neural model could be still better in order to improve studies on the nervous system, leaving aside minor adjustments, although time and the nature of things will probably modify sooner or later this impression. An eye needs to stay open in this direction to ensure that the prosomeric model does not become a centenary dogma, as happened with the columnar model. The validity of a model is limited by its utility and the credibility of its assumptions.

## Author Contributions

LP conceived and produced the work.

## Conflict of Interest

The author declares that the research was conducted in the absence of any commercial or financial relationships that could be construed as a potential conflict of interest. The handling editor declared a past co-authorship with the author LP.

## Publisher’s Note

All claims expressed in this article are solely those of the authors and do not necessarily represent those of their affiliated organizations, or those of the publisher, the editors and the reviewers. Any product that may be evaluated in this article, or claim that may be made by its manufacturer, is not guaranteed or endorsed by the publisher.
